# Electric-Driven Polarization Meta-Optics for Tunable Edge-Enhanced Images

**DOI:** 10.3390/mi13040541

**Published:** 2022-03-30

**Authors:** Cheng Cheng, Kai Ou, Hui Yang, Hengyi Wan, Zeyong Wei, Zhanshan Wang, Xinbin Cheng

**Affiliations:** 1 MOE Key Laboratory of Advanced Micro-Structured Materials, Shanghai 200092, China; 1950150@tongji.edu.cn (C.C.); 2130945@tongji.edu.cn (H.W.); weizeyong@tongji.edu.cn (Z.W.); wangzs@tongji.edu.cn (Z.W.); chengxb@tongji.edu.cn (X.C.); 2 Institute of Precision Optical Engineering, School of Physics Science and Engineering, Tongji University, Shanghai 200092, China; 3 Shanghai Frontiers Science Center of Digital Optics, Shanghai 200092, China; 4 National Research Center for High-Efficiency Grinding, College of Mechanical and Vehicle Engineering, Hunan University, Changsha 410082, China; 5 Shanghai Institute of Intelligent Science and Technology, Tongji University, Shanghai 200092, China

**Keywords:** edge-enhanced imaging, tunable metalens, polarization control

## Abstract

In this study, we demonstrate an electrically driven, polarization-controlled metadevice to achieve tunable edge-enhanced images. The metadevice was elaborately designed by integrating single-layer metalens with a liquid-crystal plate to control the incident polarization. By modulating electric-driven voltages applied on the liquid-crystal plate, the metalens can provide two polarization-dependent phase profiles (hyperbolic phase and focusing spiral phase). Therefore, the metalens can perform two-dimensional focusing and spatial differential operation on an incident optical field, allowing dynamic switching between the bright-field imaging and the edge-enhanced imaging. Capitalizing on the compactness and dynamic tuning of the proposed metadevice, our scheme carves a promising path to image processing and biomedical imaging technology.

## 1. Introduction

Integrated and efficient devices modulating optical information are highly desirable for fast and reliable large-scale image processing required in various domains such as object identification, machine vision, and artificial intelligence [1]. Optical analog computation has received extensive attention due to the unprecedented advantages of high-speed, low-power consumption, and parallel computing. Despite their capacity for optical information and image processing in real-time, traditional optical analog fashions are typically of a significantly large size [2,3]. The bulky configurations based on conventional optics hinder their applications in integrated optics and photonics.

Optical metasurfaces, planar and lightweight optical elements consisting of artificial and subwavelength resonators with tailored electromagnetic properties, have attracted extensive attention due to the possibility of powerful and flexible control over the amplitude, phase, and polarization of light [4,5,6,7,8,9,10,11,12,13,14,15,16,17,18,19,20]. Metasurface-enabled optical analog computation has made significant progress towards optical differential operations and edged-enhanced imaging through elaborately designed metasurface devices (metadevices) with a conventional 4*f* Fourier filtering system [21,22,23,24,25,26,27,28,29]. However, this inevitably introduces a complex and bulky optical system, which runs counter to the compactness of metasurfaces. Moreover, as important as edge-enhanced imaging that effectively extracts the edge information of objects, conventional bright-field imaging is able to capture the corresponding overall morphologies [30]. Therefore, developing a tunable and miniature optical system capable of switching between bright-field imaging and edge-enhanced imaging is highly desirable.

**Figure 1:**
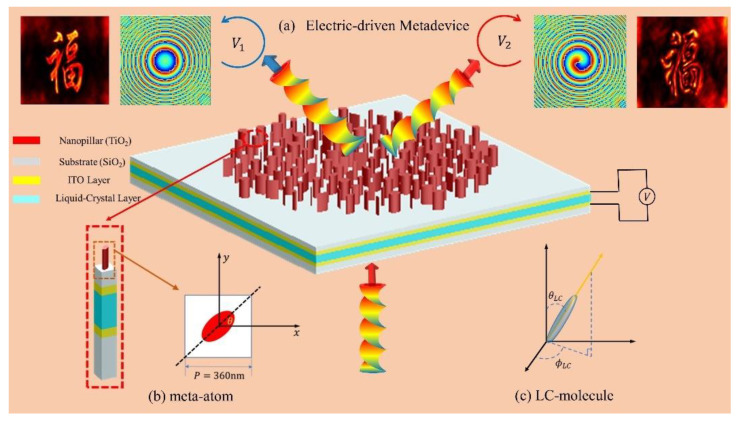
Schematic of the LC-based metadevice. (**a**) The dynamic tunable principle of the metadevices; (**b**) Schematic of the nano-pillar used in the design of the metasurface; (**c**) Schematic of the LC molecule.

An electric-driven scheme based on liquid crystals (LC) has shown great potential to enable the dynamic polarization of meta-optics, benefiting from the flexible and fast control of polarization and phase [31]. Herein, we demonstrate a compact polarization-multiplexing spiral metalens integrated with a nematic liquid-crystal (NLC) plate, which is used to control the polarization of the incident light for switching between bright-field focusing mode and the spiral phase contrast imaging mode, as shown in Figure 1. By precisely tuning the driven voltages, the polarization of the incident light can be switched between right-handed circularly polarized (RCP) to left-handed circularly polarized (LCP), which results in fast switching of the phase distributions of the LC-based metadevice. The focusing vortex phase profile of the metadevice further introduces two-dimensional spatial differentiation and converging operation to the incident filed [32]. Unlike the conventional 4*f* Fourier filtering system, the LC-based polarization-multiplexing strategy compresses the lens imaging and edge-enhancement imaging into a single-layer metalens. Benefiting from the significant advantages of being compact and integrated, in addition to the tunability of the LC-based active meta-optics, our results may find important applications in image processing.

## 2. Design and Numerical Results

Figure 1a shows the schematic and operation principle of the metadevice capable of realizing electric-driven switching between bright-field and edge-enhanced imaging. The reconfigurable metadevice consists of elliptical TiO_2_ nanopillars (n=2.48) on a SiO_2_ substrate (n=1.46). Figure 1b shows the cross-section of the nano-pillar, from which one can see the square lattice constant P=360 nm and the height of each nanopillar h=600 nm. The adjustable parameters for the elliptical nanopillars are major axis $R_{x}$, minor axis $R_{y}$, and orientation angle θ. The operation wavelength is λ=483 nm. To manipulate the circular-polarized state of the incidence, LC layer with ITO (indium tin oxide) thin layers has been integrated with the metasurface to introduce different phase delay via tuning the electric-driven voltage.

Figure 1c shows the configuration of an LC-molecule used in this work, in which the azimuthal and polar angles are denoted as $\phi_{LC}$ and $\theta_{LC}$, respectively. When introducing different applied voltage on the LC layer, the equivalent refractive index of the LC layer for *y*-linearly polarization (YLP) changes as the polar angle $\theta_{LC}$ of the LC molecule changes. The axis of the LC molecule and the electric field direction x-polarized light (XLP) are coplanar, which means the equivalent refractive index for XLP remains unchanged. Hence, the electric-driven LC layer will be able to introduce different phase delay to the incidence and enables switching between RCP and LCP states.

**Figure 2:**
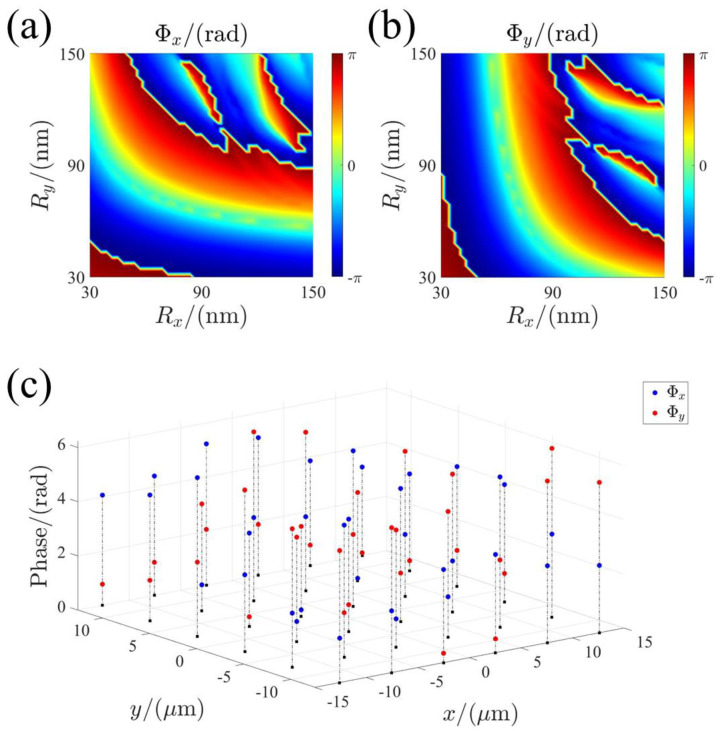
The library of the nano-pillars. (**a**,**b**) Phase shifts of the elliptical nano-pillars as a function of major axis $R_{x}$ and minor axis $R_{y}$ for *x*- and y-polarized incidences, respectively. (**c**) Phase shift for *x*- and y-polarized incidences for the selected and optimal nano-pillars used in design of the metasurface.

Figure 2a,b show the simulated phase shifts of the elliptical nano-pillars as a function of the semi-major axis and semi-minor axis under XLP and YLP incident light, respectively. Based on 3D finite-difference time-domain (FDTD) simulations, we calculate the phase shift of the nano-pillars. Periodic boundary conditions are applied along the *x* and *y* axes and perfectly matched layers (PML) are applied along the *z*–axis at the design wavelength 483 nm. Due to the symmetry, the database of y-polarization and that of x-polarization are mirror symmetry with respect to the diagonal line shown in Figure 2a,b. The phase spectra are polarization-dependent. Therefore, we can independently manipulate the phase along the different axis. For the elliptical nanopillars used in this work (shown in Figure 1), the optical response of the nano-pillars can be described by the Jones matrix as [28]:

(1) $$\;J\left(x,y\right)=R\left(-\theta \left(x,y\right)\right)\left[\begin{array}{cc} e^{i\Phi_{x}\left(x,y\right)} & 0\\ 0 & e^{i\Phi_{y}\left(x,y\right)} \end{array}\right]R\left(\theta \left(x,y\right)\right)$$

where $\Phi_{x}\left(x,y\right)$ and $\Phi_{y}\left(x,y\right)$ denote the spatial propagation phase profiles for the nano-pillar at (x,y) under *x*- and *y*-polarized incidence along two symmetry axes, θ(x,y) denotes the orientational angle of the nano-pillars, and R is a 2×2 rotation matrix which can be expressed as$\;R\left(\theta \right)=\left[\begin{array}{cc} \cos\theta & \sin\theta \\ -\sin\theta & \cos\theta \end{array}\right]$. For independent control of the phase shifts of the LCP and RCP components, the metasurface has to meet the requirement that $J\left(x,y\right)|L\rangle =e^{i\varphi_{RCP}\left(x,y\right)}|R\rangle$ and $J\left(x,y\right)|R\rangle =e^{i\varphi_{LCP}\left(x,y\right)}|L\rangle ,$ where |L〉 and |R〉. denote the LCP and RCP lights, respectively. The Jones matrix can be derived as:

(2) $$\;J\left(x,y\right)=\left[\begin{array}{cc} \frac{e^{i\varphi_{LCP}\left(x,y\right)}+e^{i\varphi_{RCP}\left(x,y\right)}}{2} & \frac{ie^{i\varphi_{LCP}\left(x,y\right)}-ie^{i\varphi_{RCP}\left(x,y\right)}}{2}\\ \frac{ie^{i\varphi_{LCP}\left(x,y\right)}-ie^{i\varphi_{RCP}\left(x,y\right)}}{2} & \frac{-e^{i\varphi_{LCP}\left(x,y\right)}-e^{i\varphi_{RCP}\left(x,y\right)}}{2} \end{array}\right]$$

where $\varphi_{LCP}$ and $\varphi_{RCP}$ denote the phase delay for LCP and RCP incident light, respectively. By combining Equations (1) and (2), polarization-decoupled phase control can be derived as:

(3) $$\;\varphi_{LCP}=\Phi_{x}-2\theta$$

(4) $$\;\varphi_{RCP}=\Phi_{y}+2\theta -\pi \;$$

(5) $$\;\theta \left(x,y\right)=\frac{\varphi_{LCP}-\varphi_{RCP}}{2}$$

To utilize edge-enhanced imaging with a single lens for LCP incidence, the phase profile of the metalens is a sum of the hyperbolic phase and the spiral phase (with topological charge of L = 1):

(6) $$\;\;\varphi_{LCP}\left(x,y\right)=\frac{2\pi }{\lambda }\left(f-\sqrt{x^{2}+y^{2}+f^{2}}\right)+L*\arctan\frac{y}{x}$$

and the phase profile of the metalens for RCP incidence can be expressed as:

(7) $$\;\varphi_{RCP}\left(x,y\right)=\frac{2\pi }{\lambda }\left(f-\sqrt{x^{2}+y^{2}+f^{2}}\right)$$

Based on Equations (3)–(7), the most appropriate set of parameters for nano-pillar at the corresponding pixel position (*x*, *y*) can be selected by minimizing an error function defined as the maximum error between the required phase and the simulated ones:

(8) $$\;Error\left(R_{x},R_{y};x,y\right)=\max\left\{\left|e^{i\Phi_{x}}-e^{i\varphi_{0}\left(x,y\right)}\right|,\;\left|e^{i\Phi_{y}}-e^{i(\varphi_{0}\left(x,y\right)+\pi )}\right|\right\}\;$$

where $\varphi_{0}\left(x,y\right)=\frac{\varphi_{LCP}\left(x,y\right)+\varphi_{RCP}\left(x,y\right)}{2}\;$.

Using the optimized filtering method, the optimal nano-pillars can be selected from the nano-pillar library (shown in Figure 2) for the polarization-multiplexing metalens. To satisfy the half-wave plate conditions derived from Equations (3)–(5), the nano-pillars with a phase shift difference of π between the x- and y-polarization can be selected, as shown in Figure 2c. Based on the proposed strategy, we have designed the polarization-multiplexing metalens.

**Figure 3:**
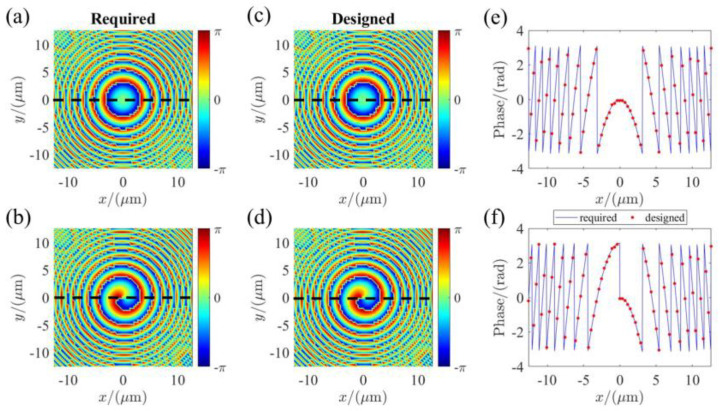
The simulated phase profiles for orthogonal circular polarizations. (**a**,**b**) are the required hyperbolic phase profile and focusing vortex phase profile, respectively; (**c**,**d**) are the optimized phase profiles offered by the nano-pillars for LCP and RCP incidence, respectively; (**e**,**f**) the corresponding phase profiles along the dashed line y=0.

Figure 3a,b show the required phase profile and the simulated ones for the metalens operating at LCP incidence (bright-field imaging mode). The corresponding phase profiles for RCP incidence (edge-enhance imaging) are shown in Figure 3c,d. As shown in Figure 3c,f, we have extracted the corresponding phase profiles along the *y*-axis (dashed lines shown in Figure 3) to further verify the validity of the design. It can be seen that the optimized phase profiles offered by the selected nano-pillars agree well with the required ones. The results indicate that the multi-functional metasurface can perform the polarization-multiplexing operation on circular-polarized incidence. Therefore, the tunable metasurface devices can be realized by introducing different phase delay (such as π and 0) on the specific circular-polarized incidence. In this work, the incidence is set as the RCP state. Benefiting from birefringence and electric-driven performance of the NLC, we can flexibly introduce different phase delays on orthogonal polarized states of the incidence by tuning electric-driven voltages. This results in fast switching between the RCP and LCP, allowing us to realize the electric-driven tunable metalens.

**Figure 4:**
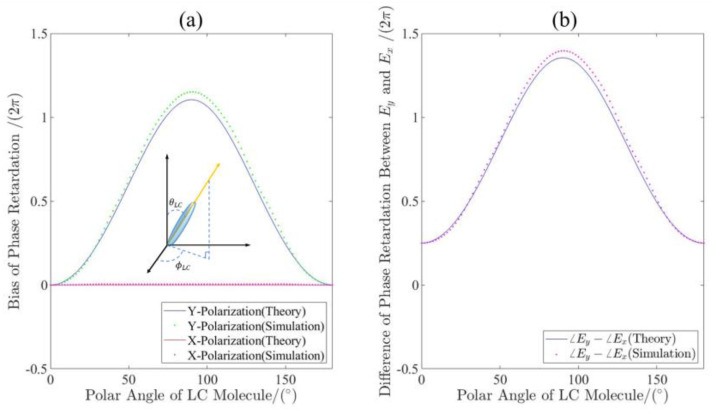
The phase retardation control of the NLC cell. (**a**) Theoretical and simulated phase retardation as functions of the polar angle of LC molecules for different incidence; (**b**) theoretical and simulated difference of phase retardation between x- and y-component of the transmitted light for LCP incidence functioning as the polar angle of LC molecule.

For proof of the concept, we have performed the design of the LC-based metalens with the diameter of 25.2 μm. The NLC cell provides dynamic polarization conversion as a tunable wave plate. The refractive index of the ordinary axis is *n_o_* = 1.529 and the extraordinary axis is *n_e_* = 1.713. The configuration of the NLC molecule (with the azimuthal angle $\phi_{LC}=90^{{}^{\circ}}$, and the polar angle *θ_LC_* = 0°) is shown in the inset of Figure 4a. If the electric-driven voltage is applied on ITO layers sandwiching the NLC cell (shown in Figure 1), the anisotropy axis of the NLC can be adjustable from in-plane to out-of-plane [31]. This results in a variable refractive index (equivalent refractive index *n*) calculated by:

(9) $$\;n=\frac{1}{\sqrt{\frac{\sin^{2}\theta_{LC}}{n_{e}^{2}}+\frac{\cos^{2}\theta_{LC}}{n_{o}^{2}}}}$$

Therefore, we can construct a tunable wave plate using the NLC. For a given wavelength, the phase retardation can be written as is $\Gamma =\frac{2\pi \left(n-n_{o}\right)}{\lambda }d$, where *d* is the thickness of the NLC layer and *λ* represents the designed wavelength. Based on FDTD methods, we have calculated the phase retardation as a function of the *θ_LC_* for XLP and YLP incidence in the transmission mode. It can be observed that the simulated results show good agreement with the theoretical ones calculated by Equation (9). Figure 4b demonstrates the calculated phase difference between the x- and y-component of the transmitted light for the LCP incidence. The results indicate that the transmitted light is LCP at the azimuthal angle $\theta_{LC}=0^{\circ }$ (the corresponding electric-driven voltage denoted as $V_{1}$) and is converted to RCP when the setting $\theta_{LC}=43.6^{\circ }$ (the electric-driven voltage denoted as $V_{2}$).

**Figure 5:**
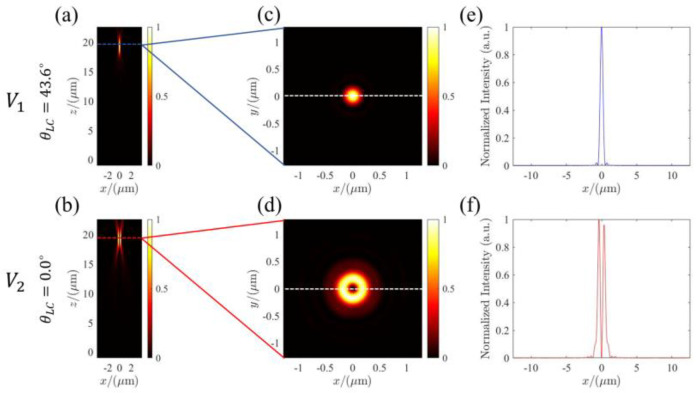
The simulations of the LC-based metalens. (**a**,**b**) Normalized intensity distribution along the x–z plan for electric-driven voltages $V_{1}$ and $V_{2}$, respectively; (**c**,**d**) the corresponding intensity distributions on focal plane at different voltages; (**e**,**f**) the corresponding x-cut distributions of the focal spots.

We further verified the performance of the LC-based metalens based on an FDTD simulation. PML boundary conditions were applied along all the three axes for the simulations with the incidence of LCP. Figure 5a,b demonstrate the longitudinal intensity profiles (along x-z plane) at the electric-driven voltages $V_{1}$ and $V_{2}$, respectively. It can be seen that solid-spot (for $V_{1}$) and hollow-shaped (for $V_{2}$) intensity profiles with the same focal length have been generated. The small deviation between the simulation and the design focal length can be explained by the discrete phase provided by the nano-pillars for the approximation of the continuous phase distribution by Equations (6) and (7).

To better analyze the characteristic of the beams, Figure 5c,d extract the focal plane intensity distributions along the dashed lines. The focal spots exhibit the electric-driven and polarization-dependent behavior (topological charge number or the beam depends on electric-driven voltage). The simulated results agree well with the theoretical expectation. The results robustly confirm the electric-driven tunable focusing property and switchable behavior of the LC-based metalens. Figure 5e,f show the corresponding x-cut intensity distributions across the focal spots (along the white dashed line) in Figure 5c,d. The full width at half maximum (FWHM) of the focal spots are 465 nm and 736 nm. The results demonstrate the nearly diffraction-limited focusing and electric-driven polarization-controlled performance of the multi-functional metalens. To the best of our knowledge, this is the first demonstration of an electric-driven multifunctional metalens based on the control of polarization. The LC-based metalens design strategy paves a promising way to active meta-optics.

**Figure 6:**
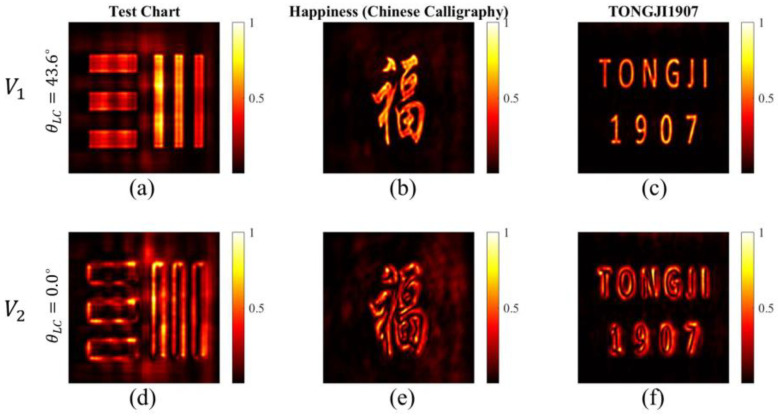
The simulated results for switching between thebright-field imaging and edge-enhanced imaging. (**a**–**c**) The bright-field images for different object with loading voltage $V_{1}$. (**d**–**f**) The corresponding edge-enhanced images with loading voltage $V_{2}$.

Based on the simulation, we extracted the point spread functions of the metalens (electrical filed distributions denoted as *E*) at different electric-driven voltages $V_{1}$ and $V_{2}$. For the focusing vortex phase profile (with loading voltage *V*_2_), the metalens can perform two-dimensional spatial differentiation and a converging operation on the incident optical filed [32]. This results in the edge-enhanced imaging effect. For our single-lens imaging system, the image field can be expressed by a convolution of the point spread function E and the object field $U_{o}$. Then, we demonstrated the dynamic switching effect between bright-field imaging and edge-enhanced imaging by using the LC-based metalens. Three different objective images (including part of the test chart, Chinese calligraphy “Fu” which means happiness, and the icon “TONGJI1907”, which means Tongji University found in 1907) were selected as typical examples. Figure 6a–c reveal the bright-field imaging property of the LC-based metalens with loading voltage V1. When switching the voltage to $V_{2}$, the LC-based metalens converts to a spiral metalens, which is able to perform two-dimensional spatial differentiation on the objective images. Figure 6a–c show the imaging property of the spiral metalens as expected; the simulated results show the two-dimensional edge-enhance imaging of the objective images. Therefore, based on the designed LC-based metalens, we have achieved an electric-driven active imaging system which shows huge superiority compared to its conventional counterpart.

## 3. Conclusions

In conclusion, we have proposed an electric-driven dynamical polarization meta-optics scheme for tunable imaging based on an LC-based polarization-controlled metalens. We have theoretically designed an electrically driven polarization-multiplexing metalens capable of achieving active switching between bright-field imaging and edge-enhanced imaging via tuning the voltage applied on the LC cell. Benefiting from the quick response of an electronic-driven liquid-crystal metasurface, the multi-functional metalens allows us tunable imaging with a fast switching speed. Compared with conventional filtering imaging systems, the electric-driven multi-functional metalens not only integrates the bright-field imaging and edge-enhanced imaging functions into a single-layer metasurface, but also compresses the imaging system into a thickness of wavelength scale. With the advantages of being compact, integrated, and having a fast tuning speed, our designed LC-based metalens may find important applications in fields such as image processing, machine vision, and artificial intelligence.

## Data Availability

The data presented in this study are available from the corresponding author upon reasonable request.
